# Application value of strain elastography in testicular injury assessment after torsion

**DOI:** 10.3389/fped.2024.1477821

**Published:** 2024-11-25

**Authors:** Jiehong Zhou, Chihan Peng, Xiaoxia Zhu, Wenqing Yao, Yan Luo, Lulu Yang

**Affiliations:** ^1^ Department of Ultrasound, West China Hospital of Sichuan University, Chengdu, China; ^2^ Department of Pathology, West China Hospital of Sichuan University, Chengdu, China

**Keywords:** testicular torsion, elastography, strain elastography, ultrasound, testes

## Abstract

**Aim:**

To evaluate the capability of strain elastography (SE) in assessing the degree of testicular injury after torsion.

**Material and methods:**

In total, 50 rabbits were divided into four groups according to different degrees of testicular torsion (TT) at 0°, 180°, 360°, and 720°. For each animal, according to the tissue stiffness distribution, an SE score and strain ratio (SR) were obtained. With the histopathological results as the reference, the correlation coefficients of the apoptotic index and SE score or SR were calculated, and the receiver operating characteristic (ROC) curves were created to assess the capability of SE in assessing the degree of testicular injury.

**Results:**

A significant positive correlation was found between the apoptotic index and SE score, as well as the SR, with corresponding correlation coefficients of 0.70 (<0.001) and 0.68 (*P* = 0.001), respectively. The areas under the ROC curves (AUCs) of the SE score and SR for identifying ischemia/hypoxia injury were found to be 0.81 (95% CI, 0.71–0.93) and 0.73 (95% CI, 0.60–0.86), respectively. For identifying irreversible damage, the AUCs were 0.69 (95% CI, 0.56–0.83) and 0.71 (95% CI, 0.59–0.84) for the SE score and SR, respectively.

**Conclusions:**

SE scores exhibited good diagnostic capability for detecting ischemia/hypoxia injury after TT. In early identification of severe injury/necrosis following TT, SE demonstrated some value but was not ideal.

## Introduction

Testicular torsion (TT) is a common pediatric scrotal emergency, accounting for 10%–15% of scrotal diseases in children (1, 2). Accurate identification and timely treatment are crucial as TT requires a different treatment approach compared to other scrotal emergencies such as orchitis and epididymitis (3–5). The salvageability of the testicles depends not only on the duration of ischemia but is also closely associated with the degree of torsion. Therefore, early diagnosis of TT and accurate assessment of the degree of testicular injury are of great clinical significance in guiding clinical intervention and selecting appropriate treatment modalities.

Color Doppler ultrasonography is the preferred imaging modality for TT evaluation in clinical emergencies. However, conventional ultrasonography heavily relies on the expertise of sonographers and the sensitivity of devices, especially in the pediatric population who have small testicles characterized by low vascular density and slow blood flow velocity (6, 7). Moreover, in some cases of incomplete torsion, the preservation of blood perfusion in the testicles may lead to a misdiagnosis and elevate the likelihood of false negatives. Contrast-enhanced ultrasound (CEUS) has been reported to be an effective method for diagnosing TT in recent years (8, 9). However, the requirement for an intravenous injection and safety concerns regarding contrast agents in pediatric patients have restricted the application of CEUS in emergency settings (10, 11).

Ultrasound elastography (UE) is a type of imaging technique that directly or indirectly reflects the softness and hardness of the tissue by using the correlation between the elastic modulus of the tissue and the biological characteristics of lesions. The current UE techniques can be classified into strain elastography (SE) or shear wave-based elastography (SWE) according to the measured physical quantity. In the SE technique, an appropriate manual compression is applied to the tissue with a transducer which works fairly well for superficial organs. In contrast, dynamic stress is applied in SWE using acoustic radiation force which can be further used to assess deeply located organs (12). It has been reported that testicular parenchyma values in SWE significantly increase after TT and that real-time SWE is an effective method for a differential diagnosis of TT (13–16). Nevertheless, in certain circumstances, SE may be the only available elastography method, especially in emergencies. Whether SE is a reliable technique for TT diagnosis and whether it can be further applied in the assessment of the degree of injury remains unclear as there are limited associated studies (17, 18).

In this study, we aimed to investigate the feasibility of using SE to estimate the degree of testicular injury following TT based on histopathological changes.

## Materials and methods

### Animals and TT modeling

In total, 50 male New Zealand rabbits weighing between 2.2 and 3.0 kg were housed in accordance with standard living conditions for a period of 2 weeks for acclimatization. The experimental protocol for this study was approved by the Animal Care and Use Committee of West China Hospital, Sichuan University (License Number: 20220519012). All the animal experiments were conducted in compliance with the National Institutes of Health’s Guide for the Care and Use of Laboratory Animals.

Rabbits were intramuscularly anesthetized with Zoletil at a dosage of 5 mg/kg, followed by intraperitoneal administration of 1% pentobarbital sodium at a dosage of 3 ml/kg. The rabbits were placed in the supine position and secured onto a tablet. The inguinal regions and scrotum were shaved and sterilized. Under sterile conditions, surgical incisions were made at a distance of 1–2 cm above the scrotum. The spermatic cord underwent a counter-clockwise rotation and was subsequently affixed to the muscular wall at the corresponding level to prevent detorsion (19). The animals were randomly assigned to group S (sham operation; *n* = 5), group A (torsion at 180°; *n* = 15), group B (torsion at 360°; *n* = 15), and group C (torsion at 720°; *n* = 15). Based on the sacrifice time after surgery (2, 4, and 6 h), the experimental animals in groups A, B, and C were further divided into three subgroups (*n* = 5 for each subgroup). Postoperative analgesia was achieved using an intramuscular administration of meloxicam at a dosage of 0.2 ml/kg.

### Strain elastogram acquisition

**Figure 1:**
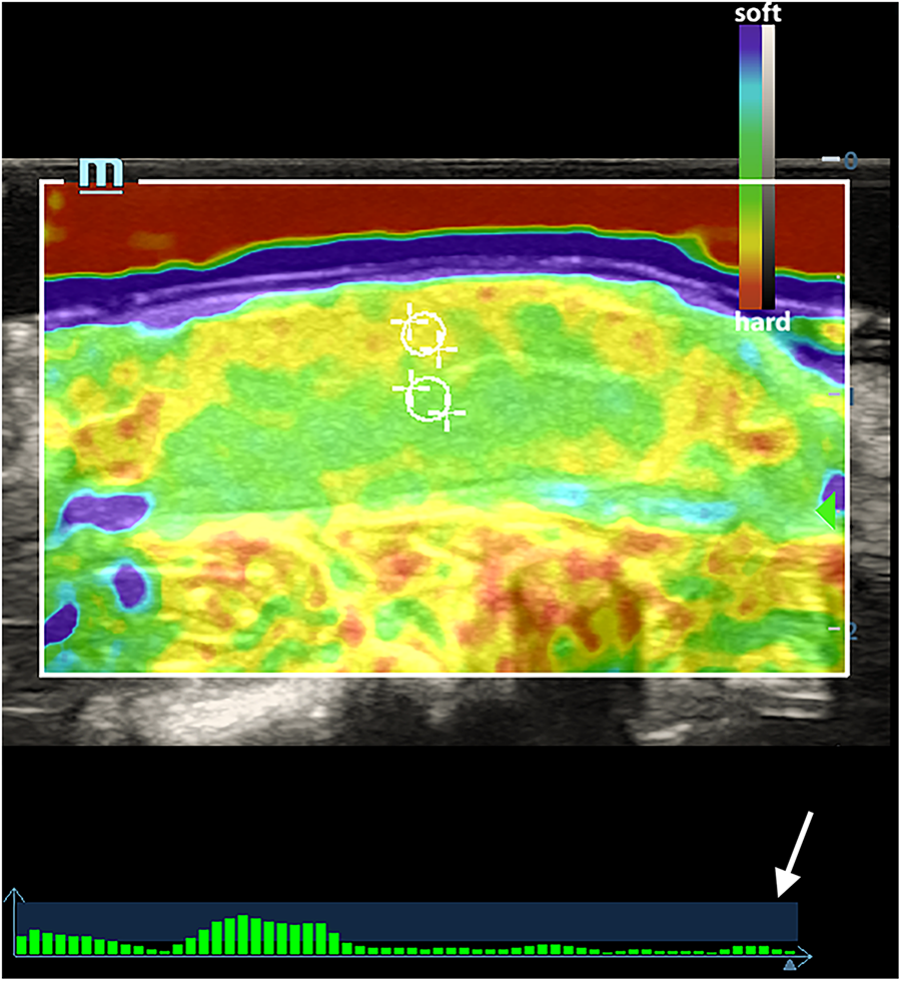
An elasticity map of a normal testicle. The white circles indicated the ROI of the testicular parenchyma beneath the capsule and in the central area. The white arrow indicates the quality feedback bar.

The elastography examinations were conducted by a sonographer with more than 8 years of experience in clinical ultrasound diagnosis and at least 5 years of experience in UE. An M9cv ultrasound system (Mindray Medical Solutions, Shenzhen, China) equipped with an elastography-compatible L12–4s transducer (frequency range of 4–12 MHz) was utilized for the stiffness assessment both pre- and post-operation. The ultrasound gel pad was placed on the surface of the testicles. The elastograms were obtained in the largest longitudinal section and the elasticity acquisition box was placed in the middle of the elastogram, including the whole testicle. Local strain was provided by the operator with manual compressions using the ultrasound transducer. A quality feedback bar was used to control the maintenance of the applied force at the bottom of the screen. The bars on the vertical axis in the blue area indicated the optimal compression. Elastography enables users to obtain tissue elasticity distribution details from intuitive images by providing 2D color imaging of tissue stiffness information. In the color-coded map, the hard tissues appear in red, soft tissues in blue to purple, and intermediate degrees of elasticity in green and yellow (Figure 1).

### Elastogram evaluation

**Figure 2:**
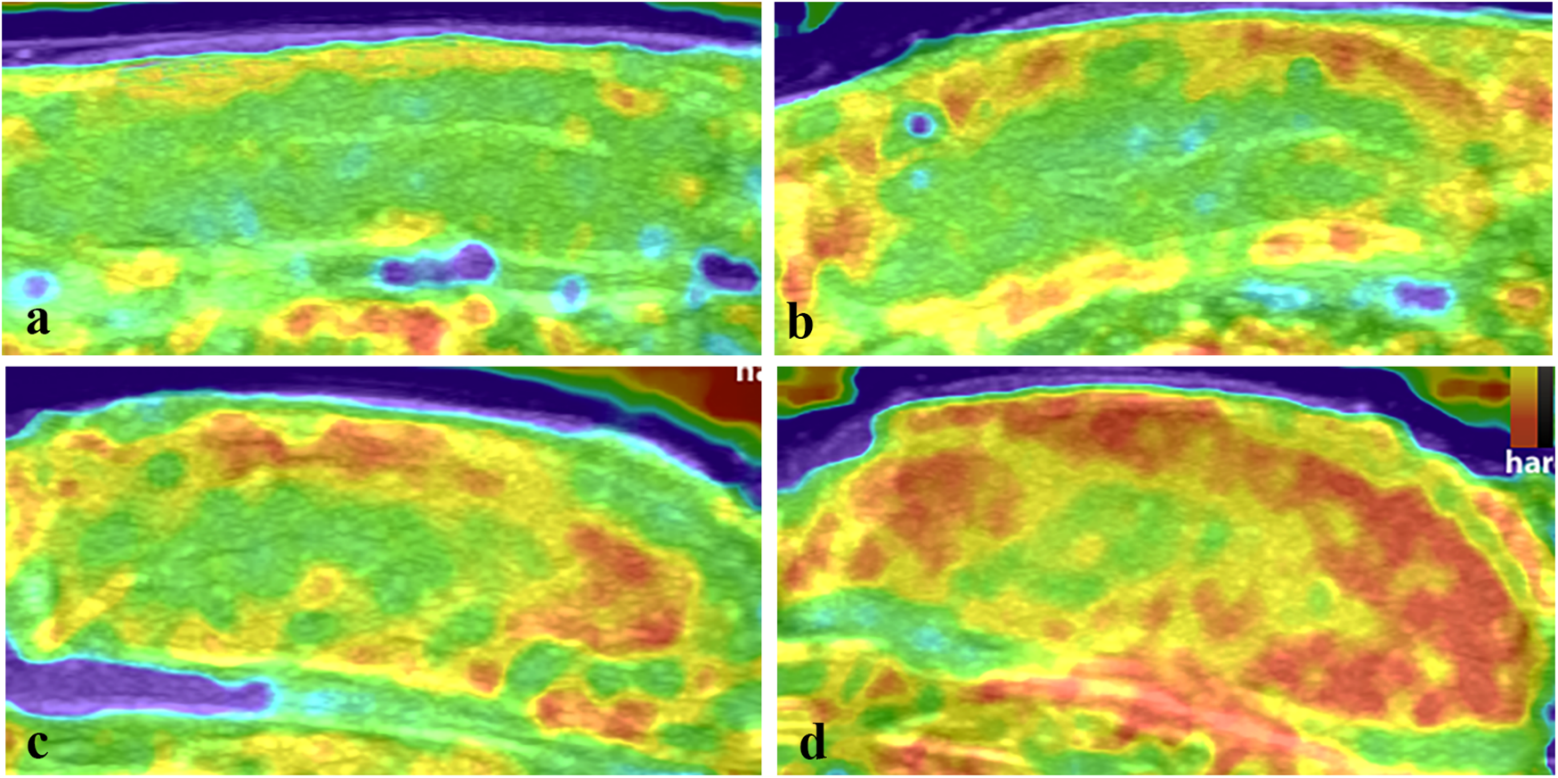
The elasticity maps of testicles in the control group and torsion groups. **(a)** Control testicles with an elastography score of 1. **(b)** Testicles after 360° torsion at 2 h post-operation, with a score of 2. **(c)** Testicles after 360° torsion at 6 h post-operation, with a score of 3. **(d)** Testicles after 720° torsion at 6 h post-operation, with a score of 4.

According to the tissue stiffness distribution indicated by different colors on the elastogram, the intratesticular elasticity was scored on a scale of 1–4 according to previous studies (18, 20, 21)^.^ Score 1: the testis is mostly soft and presents as blue and green. Score 2: the testis is moderately soft and mainly colored with yellow and green, a red area may be present but is less than 30% of the testicular parenchyma. Score 3: the testis is predominantly hard and is yellow and red but the red area accounts for no more than 70% of the testicular parenchyma. Score 4: the testis is almost entirely hard with red areas comprising 70% of the total area (Figure 2). This assessment was conducted by two radiologists with over 8 years of experience in clinical ultrasound who did not know the pathological results. When two scores were not consistent, a final score was awarded after a consensus was reached.

In the same longitudinal section, the strain ratio (SR) of the testicular parenchyma beneath the capsule and the central area was acquired by placing circle regions of interest (ROI) with a diameter of 1.5 mm in the middle line of the testicles. For each animal, five consecutive acquisitions were performed. The median value of the five SR measurements was used for further statistical analysis. The results were defined as reliable when the interquartile range/median (IQR/M) was within 30% (12, 22).

### Histopathological assessment

The testes were surgically removed at a specific time after torsion and a histological examination was further performed using hematoxylin and eosin (H&E) staining. Microscopic images of the testicular tissues were captured at magnifications of 200× and 400×. Referring to Cosentino's histological grading criteria (23, 24), the twisted testicle was classified as normal (grade 1), ischemia/hypoxia injury (grade 2 or above), or severe injury/necrosis (grades 3 and 4).

The terminal deoxynucleotidyl transferase dUTP nick end labeling method (TUNEL) was used to assess the apoptotic spermatogenic cells. A positive nucleus presented as green, while a negative one was blue under a fluorescence microscope. In total, 30 seminiferous tubules were randomly selected for the evaluation and the total number of cells and positive cells were counted. The apoptotic index was calculated for each sample using the following formula: Apoptotic index = 100 × (TUNEL-positive cells/total cells) % (25).

### Statistical analysis

The Shapiro–Wilk test was used to analyze the data distribution. The SE scores and SR were compared among different groups at different time points by one-way analysis of variance (ANOVA) with Tukey's *post-hoc* test when the data were normally distributed, while the non-parametric Kruskal–Wallis test was utilized when data were non-normally distributed. Spearman’s rank correlation was applied to evaluate the association between SE score and apoptotic index; while Pearson’s correlation coefficient was calculated to assess the association between the continuous variable SR and apoptotic index. The receiver operating characteristic (ROC) curves of SE score and SR for identifying pathological ischemia/hypoxia injury or severe injury/necrosis were created. The areas under the ROC curves (AUCs) were further calculated, and sensitivity and specificity were determined using the optimal cutoff points that maximized the Youden index (26). SPSS (version 26.0, SPSS, Chicago, IL, United States) and GraphPad software (version 9.2, GraphPad Software, San Diego, CA, USA) were used for the statistical analyses and graphs. Differences were considered significant when the *P*-values were <0.05.

## Results

### Histopathological changes

**Figure 3:**
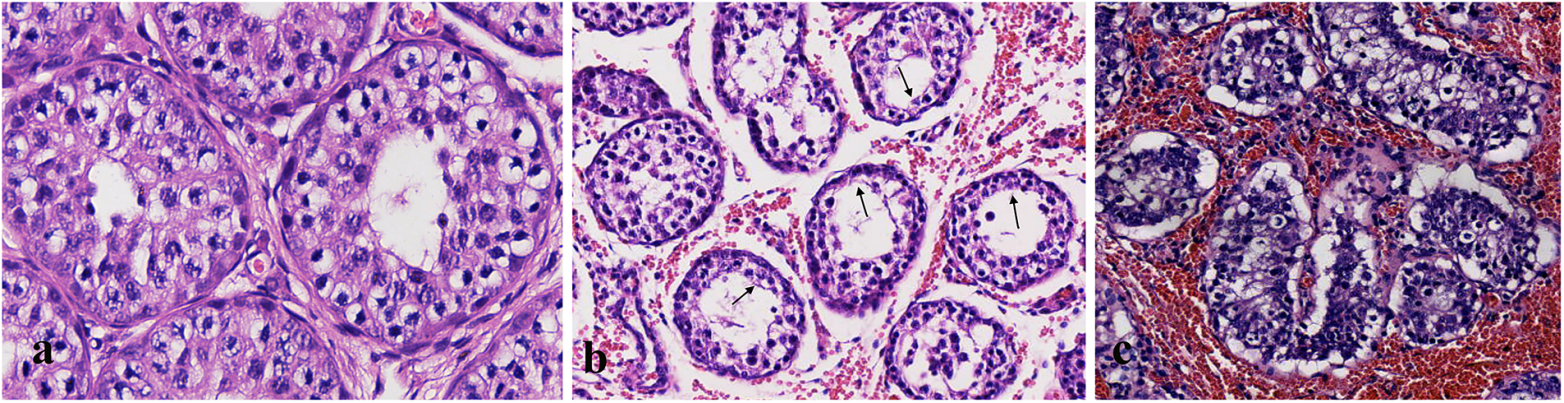
Histopathological changes of the testicles in the sham group and torsion groups. **(a)** Testicles in the control group. **(b)** Interstitial hemorrhage and reduction of the spermatogenic epithelium (black arrow) were observed in the 360° torsion group at 2 h post-operation. **(c)** Severe injury/necrosis was present in the 720° testicular torsion group after 6 h following operation, with extensive interstitial hemorrhage and seminiferous tubule destruction. (Original magnification of ×200).

The sham-operated testicles in group S showed normal testicular structure with orderly arranged spermatogenic cells and mature spermatozoa (Figure 3a). In contrast, 36 of the 45 (80%) twisted testicles exhibited ischemia/hypoxia injuries, which presented as varying degrees of interstitial hemorrhage and reduction of the spermatogenic epithelium (Figure 3b). Moreover, 18 (40%) of the testicles exhibited severe injury/necrosis, which was characterized by destroyed bound seminiferous tubules, disordered sloughed cells in the lumen, and nucleus pyknosis (Figure 3c).

### The changes in SE scores and SR

As shown in Figure 1, normal testicle parenchyma is predominantly green and blue in SE, with the skin and capsule area in yellow. After 360° and 720° TT, the central and border areas of the parenchyma in the twisted testicles were an uneven yellow or yellow-red color. Some of the twisted cases were red in color with a “stiff ring sign” in the capsule area. The SE scores in different groups at various times are shown in Table 1.

**Table 1:** The strain elastography scores for different degrees of testicular torsion at different time points.

Group	Pre	2 h	4 h	6 h
S	1.00 ± 0.00	1.20 ± 0.45	1.20 ± 0.45	1.00 ± 0.00
A	1.27 ± 0.59	1.60 ± 0.55	2.00 ± 0.71**	2.00 ± 0.71**
B	1.07 ± 0.26	1.40 ± 0.55	2.40 ± 0.55**	2.60 ± 0.55*^,^**
C	1.20 ± 0.41	1.80 ± 0.45	2.40 ± 0.55**	2.60 ± 0.89*^,^**

Group S: sham operation; group A: 180° testicular torsion; group B: 360° testicular torsion; group C: 720° testicular torsion.

* *P* < 0.05 vs. group S.

** *P* < 0.05 vs. pre-operation.

The SR of the testicle parenchyma beneath the capsule and the central area is given in Table 2. For normal testes, the stiffness under the capsule was slightly higher than that of the central area with an SR of approximately 1.2. With the increase in torsion degree and duration of ischemia, the SR slightly increased and significant differences were observed in the 360° and 720° TT groups at 6 h after the operation when compared with the sham group.

**Table 2:** The strain ratio for different degrees of testicular torsion at different time points.

Group	Pre	2 h	4 h	6 h
S	1.26 ± 0.10	1.21 ± 0.09	1.23 ± 0.11	1.19 ± 0.07
A	1.24 ± 0.98	1.27 ± 0.24	1.34 ± 0.13	1.45 ± 0.12
B	1.25 ± 0.10	1.42 ± 0.13	1.60 ± 0.14*^,^**	1.59 ± 0.26*^,^**
C	1.22 ± 0.96	1.26 ± 0.18	1.41 ± 0.18	1.60 ± 0.26*^,^**

Group S: sham operation; group A: 180° testicular torsion; group B: 360° testicular torsion; group C: 720° testicular torsion.

* *P* < 0.05 vs. group S.

** *P* < 0.05 vs. pre-operation.

### The correlation between SE score or SR and apoptotic index

**Figure 4:**
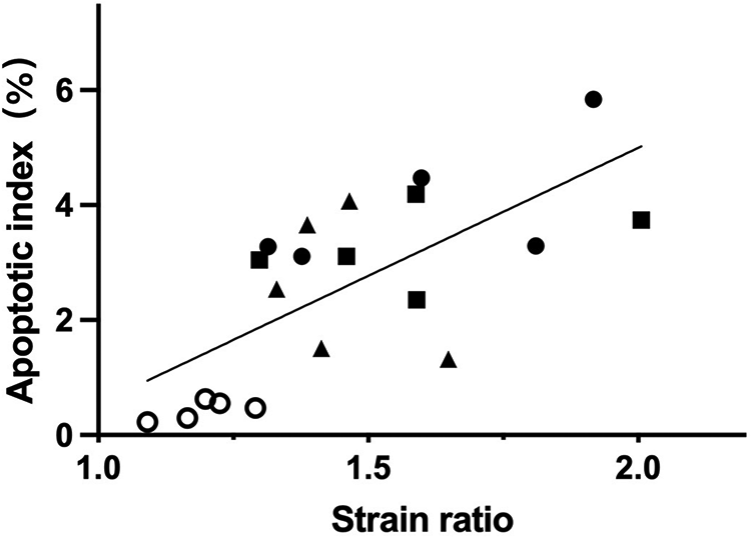
At 6 h after operation, the SR and the apoptotic index of spermatogenic cells had a positive correlation (*r* = 0.68, *P* = 0.001). The circle, triangle, square, and dot indicate the sham group and the testicular torsion at 180°, 360° and 720° groups, respectively.

At 6 h after the surgery, a positive correlation was observed between the SE score and apoptotic index. Spearman’s correlation coefficient was 0.70 (*P* < 0.001). Furthermore, as shown in Figure 4, a significant positive correlation was also found between the SR and apoptotic index, with a Pearson’s correlation coefficient of 0.68 (*P* = 0.001).

### The diagnostic performance of elastography for identifying ischemia/hypoxia injury after TT

Regarding the histopathological changes that occurred after TT, the AUCs of the SE score and SR for identifying ischemia/hypoxia injury were found to be 0.81 (95% CI, 0.71–0.93) and 0.73 (95% CI, 0.60–0.86), respectively. With a cutoff value of 2 for the SE score, the corresponding specificity was 100%, as shown in Table 3.

**Table 3:** The diagnostic capability of strain elastography for identifying ischemia/hypoxia injury after testicular torsion.

Variable	AUC (95% CI)	Cutoff value	Sensitivity (%)	Specificity (%)
SE score	0.81 (0.71–0.93)	2	48.5	100
SR	0.73 (0.60–0.86)	1.31	78.8	63.0

### The diagnostic performance of elastography for identifying severe injury/necrosis after TT

The AUCs of the SE score and SR for identifying irreversible damage were 0.69 (95% CI, 0.56–0.83) and 0.71 (95% CI, 0.59–0.84), respectively (Table 4). The diagnostic sensitivity of the SE score and SR were both greater than 80% when distinguishing testicles that had suffered severe injury/necrosis from those that had not. However, the specificity was not ideal.

**Table 4:** The diagnostic capability of strain elastography for identifying severe injury/necrosis after testicular torsion.

Variable	AUC (95% CI)	Cutoff value	Sensitivity (%)	Specificity (%)
SE score	0.69 (0.56–0.83)	1	82.3	47.6
SR	0.71 (0.59–0.84)	1.31	83.3	52.4

## Discussion

The testicular capsule is composed of a visceral layer comprising the tunica vaginalis, tunica albuginea, and a vascular membrane. The tunica albuginea is a tough fibrous covering, while the parenchyma is soft and is mainly composed of seminiferous tubules. After TT, a rotated vascular pedicle results in an obstruction of arterial inflow and venous drainage. With a greater duration of torsion time, the swollen testis will further compress the tunica albuginea and the hardness will increase, especially in the parenchyma beneath the capsule. Furthermore, a prolonged duration of venous stasis may lead to a microthrombus and eventual infarction. All these pathological changes result in increased hardness of the testicular parenchyma, and the subcapsular stiffness is significantly increased which presents as a “stiff ring sign” on an elastogram (13, 14, 27).

The investigations into using SE in diffuse testicular disease diagnosis are very limited (18, 28–30), and rarely focus on injury assessment after TT. In this study, we observed that the SR in the experimental group exhibited an increasing trend with the duration of torsion time and the progression of torsion degree. At 6 h after the operation, the SR of the 360° and 720° torsion groups was approximately 1.60, which was significantly higher than the pre-operation SR. Regarding the color distribution on the elastogram, it was confirmed that although the hardness of both the subcapsular and central parenchyma increased after TT, the hardness of the subcapsular parenchyma was significantly increased compared to that of the central area. Similar results were also observed in previous investigations (13–16). Xue et al. (13) found that the SWE value of the subcapsular parenchyma after TT was 138.76 ± 58.27 kPa, while the elasticity value of the central testicular parenchyma was 12.39 ± 6.56 kPa.

For situations where the degree and duration of torsion are unknown, the SE score showed good diagnostic capability in detecting ischemia/hypoxia injury with an AUC of 0.81 and the corresponding specificity was 100%. Thus, the SE score could indicate the possibility of parenchyma injury caused by torsion, and a prompt clinical intervention should be made. However, when discriminating between testicles that had suffered severe injury/necrosis and those that had not, SE exhibited fair diagnostic accuracy with an AUC of 0.69 and 0.71. The corresponding sensitivity was above 80%, while the specificity was below 60%. In this situation, the degree of injury may be overestimated. It should be mentioned that at 6 h post-operation, both SE score and SR demonstrated a significant positive correlation with the apoptotic index, which shows that SE is promising for identifying irreversible necrosis after TT in the long term. Hence, for the early identification of necrosis following TT, SE exhibits some value but is not ideal.

Lin et al. (14) verified that Young's modulus measured by SWE was positively correlated with intratesticular pressure. However, we barely found any obvious interstitial fibrosis in the experimental groups under a light microscope. Therefore, we speculated that in the acute stage of TT, the testicular elastograms mainly indicated the changes in intratesticular pressure and the degree of interstitial edema, instead of reflecting the status of interstitial fibrosis and necrosis. This may be one of the reasons why SE showed good performance in the early detection of mild injury following TT and was limited when discriminating severe injury/necrosis in the acute stage.

Several limitations in our study should be mentioned. First, we only focused on the role of SE in TT assessment within the recommended 6-h treatment window, thus, whether SE is still applicable in the long term needs further investigation. Second, the number of animals we investigated is comparatively small and the diagnostic cutoff values need to be further investigated in a study with a larger sample size. Finally, this is only a preliminary study, and additional research on humans is required to examine the diagnostic potential of SE for assessing the degree of testicular injury after TT.

To summarize, SE scores exhibited good diagnostic capability for detecting ischemia/hypoxia injury after TT. However, while SE demonstrated some value for the early identification of severe injury/necrosis following TT, it was not ideal.

## Data Availability

The raw data supporting the conclusions of this article will be made available by the authors, without undue reservation.
